# Presence of post-COVID-19 condition in six ethnic groups in Amsterdam, the Netherlands: a retrospectively reconstructed time interval approach

**DOI:** 10.1017/S095026882610199X

**Published:** 2026-10-14

**Authors:** Merve Spronk, Anders Boyd, Sophie Louise Campman, Vita Jongen, Henrike Galenkamp, Felix Chilunga, Charles Agyemang, Janke Schinkel, Elke Wynberg, Karien Stronks, Maria Prins

**Affiliations:** 1 Infectious Diseases, Amsterdam UMC Location AMC, Netherlands; 2 Department of Infectious Diseases, Public Health Service of Amsterdam, Netherlands; 3 Infectious Diseases, Amsterdam Institute for Infection and Immunity, Netherlands; 4 Stichting HIV Monitoring, Netherlands; 5 Department of Public and Occupational Health, Amsterdam UMC Location AMC, Netherlands; 6 Health Behaviors and Chronic Diseases, Amsterdam Public Health Research Institute, Netherlands; 7 Department of Medicine, The Johns Hopkins University School of Medicine, USA; 8 Department of Medical Microbiology and Infection Prevention, Amsterdam UMC Location AMC, Netherlands; 9 Faculty of Tropical Medicine, Mahidol Oxford Tropical Medicine Research Unit, Thailand

**Keywords:** ethnic minority groups, health disparities, long COVID, post-acute COVID-19 condition, post-acute COVID-19 syndrome, ethnicity, post COVID

## Abstract

In this retrospective cohort study, we assessed the prevalence of post-COVID-19 condition (PCC) and its determinants among six ethnic groups in Amsterdam, the Netherlands. We analysed data from participants of the HELIUS cohort with SARS-CoV-2 infection who answered questions on the presence and duration of COVID-19-related symptoms between May and November 2022. We estimated the prevalence of PCC, defined as having ≥1 of 15 pre-determined COVID-19-related symptoms remaining for at least 3 months after infection, by ethnicity at several timepoints since infection adjusted for age and sex, and assessed determinants of PCC overall using logistic regression. Of 932 infected participants, 31% [95% confidence interval (95%CI): 27%–37%] developed PCC. At 3 months after infection the adjusted prevalence of PCC varied from 17% (95%CI: 13%–24%) in the Dutch-origin group to 41% (95%CI: 34%–48%) in the South-Asian Surinamese group, and was significantly higher among participants of South-Asian Surinamese, Turkish and Moroccan origin compared to those of Dutch origin (all *P* < 0.001). Participants who were of South-Asian Surinamese origin, older, female, without a vocational education or university degree, or obese were more likely to have PCC. Those of Ghanaian origin or infected during the period of Omicron predominance were less likely to have PCC. There were clear between-ethnic differences in the prevalence of PCC, which was more common in individuals of South-Asian Surinamese, Turkish, and Moroccan origin compared to those of Dutch origin. These differences mirror ethnic differences in SARS-CoV-2 infection and disease severity. Certain factors of PCC emerged, while consistent across ethnic groups.

## Introduction

A substantial proportion of individuals exposed to severe acute respiratory syndrome coronavirus 2 (SARS-CoV-2) are known to experience long-term health effects months after infection, referred to as post-COVID-19 condition (PCC) or long COVID [1]. More than 65 million individuals worldwide were estimated to have PCC in 2023 [2]. PCC is a complex and multisystem condition, defined by the World Health Organization as the continuation or development of new symptoms 3 months after initial SARS-CoV-2 infection without any other underlying cause, with these symptoms lasting for at least 2 months [1].

Early in the COVID-19 pandemic, it became clear that ethnic minority populations were at increased risk of SARS-CoV-2 infection and severe disease outcomes compared to the majority population [3–5]. It could then be hypothesized that PCC would follow similar ethnic differences, yet the few studies investigating this question have shown contrasting results. A study from the United Kingdom found a lower risk of PCC in primary care among individuals with a Black or South Asian ethnicity compared to individuals with white ethnicity [6]. In contrast, studies from the Netherlands, Denmark and Sweden have demonstrated an increased risk of PCC in specific ethnic minority groups [7–9]. However, these studies only captured data from individuals with PCC who were hospitalized [7, 8] or with registry-based PCC in outpatient settings (excluding general practitioners) [9]. In addition, the Dutch study was not able to adjust for socioeconomic status, which may partly explain differences between ethnic minorities and the ethnic-majority population [10]. Given that the majority of individuals with COVID-19 were asymptomatic or experienced only mild symptoms, a population-based assessment that includes individuals with mild COVID-19 and accounts for socioeconomic status would provide a more comprehensive understanding of ethnic differences in the development of PCC, capturing the broader, community-level burden of PCC that may otherwise remain underdiagnosed or overlooked in clinical settings.

We therefore leveraged data from a multi-ethnic, population-based cohort study of individuals participating before the start of and during the COVID-19 pandemic to assess the prevalence of PCC and its determinants between six ethnic groups living in Amsterdam, the Netherlands. We also evaluated the prevalence of specific symptoms involved with PCC over time since infection between ethnic groups.

## Methods

### Study design and population

The Healthy Life in an Urban Setting (HELIUS) study is a population-based multi-ethnic prospective cohort study from Amsterdam, the Netherlands, which focuses on the causes of potential ethnic disparities in cardiovascular disease, mental health, and infectious diseases. Study procedures have been described previously [11].

Briefly, individuals were randomly sampled from the municipality registry of Amsterdam, after stratification by ethnicity, and invited to participate. At baseline 24 780 participants of Dutch, Surinamese, Ghanaian, Moroccan, and Turkish origin were included between January 2011 and December 2015 [11]. Ethnicity was defined according to the country of birth of the participant and their parents [11]. Participants were considered to have a migration background if (i) they were born abroad and had at least one parent born abroad (first-generation) or (ii) they were born in the Netherlands but both their parents were born abroad (second-generation). Participants born in the Netherlands and of whom both parents were also born in the Netherlands were considered as Dutch origin. After data collection, participants with a Surinamese background were further classified into African, South-Asian, Javanese, or ‘other’ based on self-report during the baseline visit [11]. Between May 2019 and November 2022, all baseline participants who were still alive according to the municipality population register and living in the Netherlands were invited for the first follow-up visit. Follow-up HELIUS data were collected among 11 035 participants [12].

A longitudinal substudy on the epidemiology and impact of COVID-19 at the population level was initiated shortly after the start of the pandemic, consisting of three subsequent study visits embedded in the parent cohort [13]. Inclusion and exclusion criteria are described in Supplementary Figure S1. The first COVID-19 substudy visit took place between June 24 and October 9, 2020. Participants of the first COVID-19 substudy visit were invited to participate in the second COVID-19 substudy visit between November 23, 2020 and June 4, 2021 and the third COVID-19 substudy visit between May 17 and November 21, 2022. To reduce selection bias, during the third substudy visit any participant who could not attend the study visit due to PCC was visited at home.

In this study, we included participants who completed the third COVID-19 substudy visit and reported ever having SARS-CoV-2 infection.

The study complies with the Declaration of Helsinki and was approved by the Amsterdam Medical Center Ethical Review Board (MREC 10/100# 17.10.1729; amendment approval for SARS-CoV-2 serological substudy: MREC 2010_100#B20203). Written informed consent was obtained from all participants.

### Study procedures

During all study visits, data were obtained through questionnaires/interviews and physical examinations, during which biological samples were obtained.

Age and sex were derived from the municipal registry. The baseline questionnaires contained questions about socio-demographic characteristics (e.g., education level, migration history), health-related behaviours (e.g., smoking), and underlying health conditions (e.g., history of cardiometabolic and cardiovascular diseases). The physical examination at baseline included anthropometry and blood pressure measurements. The measurements included during the first follow-up were largely similar to those at the baseline visit [12].

During the three COVID-19 substudy visits, participants completed questionnaires including items on SARS-CoV-2 exposure, testing, infection history, vaccination and COVID-19-related symptoms [14]. Blood samples were obtained via venepuncture, stored at −20°C, and were tested for SARS-CoV-2-specific spike protein antibodies using the WANTAI SARS-CoV-2 antibody enzyme-linked immunosorbent assay (ELISA) (Wantai Biological Pharmacy Enterprise Co., Beijing, China), according to the manufacturer’s instructions [15]. Participants were informed about their test results.

### Outcome assessment

The primary outcome of this study was the prevalence of PCC, which was defined as having one or more COVID-19-related symptoms remaining for at least 3 months after SARS-CoV-2 infection [1]. All information used to define the primary outcome was self-reported.

During the third COVID-19 substudy visit, participants who reported a prior SARS-CoV-2 infection were asked if they had suffered any symptoms from the onset of infection, and if so, how long these symptoms had persisted (i.e., less than a week, 1–4 weeks, 1–3 months, 4–6 months, 7–12 months, 1–2 years, more than 2 years) and whether these symptoms were still present at the time of study visit. These questions were asked for each of the 15 COVID-19-related symptoms – fever, cough, throat pain, fatigue, nasal cold, shortness of breath, muscle pain, loss of smell/taste, loss of appetite, headache, impaired concentration, memory complaints, confusion, difficulty with daily activities, and hair loss. If the participant had more than one prior SARS-CoV-2 infection, this question referred to the most severe SARS-CoV-2 infection (Supplementary methods). The responses to these questions were used to determine if persistent symptoms/PCC were present at reconstructed timepoints after SARS-CoV-2 infection (i.e., months 1, 3, 6, 12, and 24 months).

### Covariables

We used the following data from the baseline visit of the HELIUS study: age (recalculated for age at the third COVID-19 substudy visit and age at SARS-CoV-2 infection; categorized as <45 years, 44–54 years, 55–59 years, and ≥60 years, based on quartiles and in line with previous publications of the COVID-19 substudy [14, 16]), sex, ethnicity, educational level (higher vocational and university education versus other), indicators for access to health (i.e., Dutch language proficiency, subjective health literacy), and cultural orientation [no integration (including separation and marginalization) versus integration (also including assimilation)]. Health literacy was measured using the validated set of brief screening questions (SBSQ) questionnaire [17] and categorized as adequate or inadequate. Dutch language proficiency was categorized as difficulty with the Dutch language or not. Detailed information on the instruments used has been previously described [16].

We used the following data from either the baseline or follow-up HELIUS visit, whichever occurred closest to and before SARS-CoV-2 infection: body mass index (BMI), smoking and comorbidities [e.g., history of cardiovascular disease, diabetes mellitus, asthma/chronic obstructive pulmonary disease (COPD)]. BMI was categorized into three groups: healthy weight (BMI <25 kg/m^2^), overweight (BMI ≥25 and < 30 kg/m^2^), and obese (BMI ≥30 kg/m^2^). Smoking was categorized into three groups: non-smoker, former smoker, and current smoker. Comorbidities were based on measurements, self-report or medication use for diabetes mellitus, asthma/COPD, hypertension, and cardiovascular disease, as previously described [16]. These comorbidities have been described as possible risk factors for PCC [6, 7, 18].

From the third COVID-19 substudy visit, we used the following data: COVID-19 self-report of past SARS-CoV-2 infections, including the number and the most severe infection, hospital and ICU admission due to the SARS-CoV-2 infection, SARS-CoV-2 vaccination status, labour force participation at the time of infection, and sick leave within the first 4 weeks following infection. Vaccination status was categorized into two groups: vaccinated and non-vaccinated. Calendar period of infection was categorized into four categories to reflect the dominant variant circulating in the Netherlands: pre-alpha period (January 2020–February 2021), alpha variant (March–June 2021), delta variant (July–December 2021) and omicron variant (January–October 2022) [19]. We assumed that participants infected during the pre-alpha period were not vaccinated [20]. Sick leave within the first 4 weeks following infection was categorized for participants employed at the time of infection into: no sick leave, sick leave <10 days, and sick leave ≥10 days.

### Statistical analysis

We used an approach whereby we reconstructed longitudinal follow-up from cross-sectional data. Here, we used the date at which the questionnaire was filled out, i.e., the third substudy visit, as the last ‘follow-up’ date. The time-frame between when SARS-CoV-2 infection (or the most severe infection if there were multiple infections) began, month 0, and the last ‘follow-up’ date allowed us to assign a follow-up visit timepoint for this last date (i.e., months 1, 3, 6, 12, or 24). Based on the responses to symptom questions, we imputed whether symptoms were present or absent for this last and each of the preceding visit timepoints. This construction rendered it possible to calculate the prevalence of PCC at months 1, 3, 6, 12, and 24 from infection date, with individuals dropping out when they reached their last reconstructed follow-up visit. Of note, not all individuals had data until the 24 month reconstructed follow-up visit.

Participant characteristics were summarized as median [interquartile range (IQR)] for continuous variables and as counts (percentages) for categorical variables. We visualized the proportion of each COVID-19-related symptom by ethnic group at months 3 and 12 using radar plots.

We estimated the prevalence of any and individual persistent SARS-CoV-2-related symptoms, along with their 95% confidence intervals (CIs), during follow-up using logistic regression models with reconstructed time intervals as a covariate and a robust covariance matrix to ensure that the variance was independent and identically distributed across participants. An interaction term between interval time and ethnicity was added as a covariate to assess ethnic differences in symptom prevalence over time. These models were adjusted for age (continuous) and sex. *P*-values were obtained using the Wald χ^2^ test.

We used the logistic regression model above to assess determinants of PCC over time since infection. We initially examined the univariable associations of the following covariables on PCC: ethnicity, age, sex, education level, health literacy, labour force participation, smoking, obesity, comorbidities, SARS-CoV-2 vaccination status, and calendar period of SARS-CoV-2 infection. We included a variable combining calendar period of infection and vaccination status to address the overlap between these two variables. We constructed a multivariable model by including all covariables associated with a *P*-value <0.2 from a Wald χ^2^ test in univariable analysis. We then removed all covariables that did not significantly improve model fit (*P* > 0.05) based on the Wald χ^2^ test in backwards-stepwise fashion to arrive at a final multivariable model (model 1). We used the *P*-value from Type II Wald χ^2^ test to assess model fit when adding each covariate to the multivariable model. We forced ethnicity, age, and sex in all multivariable models during covariable selection. We calculated the adjusted odds ratios (aORs) and their corresponding 95% CIs comparing the odds of PCC across levels of determinants from the final multivariable model. We additionally tested for interaction between ethnicity and significant determinants one-by-one in separate models. We excluded participants from the model if they had missing data on any of the model covariables.

In post hoc analyses, we examined the influence of COVID-19 disease severity on ethnic differences in PCC in the multivariable model (model 2). Since we did not have data on the severity of the acute phase of infection and the number of hospitalization or admittance to intensive care was small, we included the covariable sick leave due to infection within the first 4 weeks following infection as a proxy of disease severity. For this model, we excluded participants who were unemployed or were already on sick leave at the time of infection. Second, we examined the influence of covariables that specifically applied to individuals with a migration background (i.e., migration generation, Dutch language proficiency, cultural orientation) among the potential determinants (model 3). We excluded individuals of Dutch origin for this model.

In a sensitivity analysis, we assessed the effect of additionally including unknown infections (i.e., no self-reported infection despite positive SARS-CoV-2 antibody test at the first or second substudy visits) on prevalence estimates and the multivariable model. Since vaccination campaigns were not fully implemented during these visits, we assumed that a positive spike protein antibody test result would reflect infection and not vaccination. We used the date of the substudy visit with a positive SARS-CoV-2 antibody test as the date of SARS-CoV-2 infection for these analyses. We assumed that no symptoms occurred in the individuals with unknown infections.

A *P*-value <0.05 was considered statistically significant. All statistical analyses were performed using R (version 4.4.2, Vienna, Austria).

## Results

### Description of the study population

Between May and November 2022, 1505 (61%) individuals participated in the third COVID-19 substudy visit. Of them, we excluded 562 (37%) who did not report ever having SARS-CoV-2 infection and 11 (0.1%) who did not answer the question on infection. In total, 932 participants were included in the analysis (Supplementary Figure S1). Characteristics of included and excluded participants are shown in Supplementary Table S1.

Of the 932 participants included, 724 (78%) reported only ever having one SARS-CoV-2 infection. Few participants reported being hospitalized (*n* = 23, 3%) or admitted to the intensive care unit because of COVID-19 (*n* = 9, 1%). Of the 657 participants who were employed and not on sick leave before SARS-CoV-2 infection, 206 (22% of the 932 participants) and 225 (24%) had sick leave <10 and ≥10 days, respectively, while the remaining 226 (24%) did not take sick leave in the first 4 weeks following infection.

Most individuals were of Dutch origin (*n* = 229, 25%), followed by those of South-Asian Surinamese (*n* = 195, 21%), African Surinamese (*n* = 170, 18%), Turkish (*n* = 143, 15%), Moroccan (*n* = 142, 15%), and Ghanaian (*n* = 53, 6%) origin (Table 1). Participants of Dutch origin were older, more likely to have a higher vocational or university degree, more likely to have acquired SARS-CoV-2 in the period when Omicron was the dominant circulating variant of interest, and more likely to be vaccinated against SARS-CoV-2; they were less likely to be overweight or obese than the other ethnic groups (Tables 1 and 2). Those of Turkish and Moroccan origin had a higher number of prior SARS-CoV-2 infections than the other groups.

**Table 1.** Characteristics of the HELIUS participants in the COVID-19 substudy, overall and per ethnic group, Amsterdam, the Netherlands, May 17, 2022 to November 21, 2022 (*n* = 932) Table 1. long description.

Characteristic	Total (*n* = 932)	Dutch (*n* = 229)	South-Asian Surinamese (*n* = 195)	African Surinamese (*n* = 170)	Ghanaian (*n* = 53)	Turkish (*n* = 143)	Moroccan (*n* = 142)	*P* value^a^
	*n* (%)	*n* (%)	*n* (%)	*n* (%)	*n* (%)	*n* (%)	*n* (%)	
Sociodemographic factors								
Age in years, median (IQR)^b^	55 (45, 62)	59 (48, 68)	55 (45, 62)	58 (49, 64)	55 (47, 61)	49 (43, 57)	50 (43, 56)	<0.001
<45	233 (25.0)	47 (20.5)	47 (24.1)	26 (15.3)	13 (24.5)	48 (33.6)	52 (36.6)	<0.001
45–54	249 (26.7)	44 (19.2)	53 (27.2)	41 (24.1)	14 (26.4)	49 (34.3)	48 (33.8)	
55–59	158 (17.0)	35 (15.3)	36 (18.5)	36 (21.2)	8 (15.1)	20 (14.0)	23 (16.2)	
≥60	292 (31.3)	103 (45.0)	59 (30.3)	67 (39.4)	18 (34.0)	26 (18.2)	19 (13.4)	
Sex^c^								0.050
Male	367 (39.4)	102 (44.5)	67 (34.4)	54 (31.8)	25 (47.2)	62 (43.4)	57 (40.1)	
Female	565 (60.6)	127 (55.5)	128 (65.6)	116 (68.2)	28 (52.8)	81 (56.6)	85 (59.9)	
Education level^c^								<0.001
No higher vocational or university education	580 (63.4)	76 (33.2)	151 (77.4)	113 (66.5)	44 (88.0)	95 (69.9)	101 (74.8)	
Higher vocational or university education	335 (36.6)	153 (66.8)	44 (22.6)	57 (33.5)	6 (12.0)	41 (30.1)	34 (25.2)	
Missing	17 (1.8)	0 (0.0)	0 (0.0)	0 (0.0)	3 (5.7)	7 (4.9)	7 (4.9)	
Migration generation^c^								0.020
1^st^ generation	556 (79.1)	NA	149 (76.4)	141 (82.9)	50 (94.3)	108 (75.5)	108 (76.1)	
2^nd^ generation	147 (20.9)	NA	46 (23.6)	29 (17.1)	3 (5.7)	35 (24.5)	34 (23.9)	
Employment status before infection^d^								0.605
Paid employed	657 (70.5)	164 (71.6)	135 (69.2)	123 (72.4)	42 (79.2)	96 (67.1)	97 (68.3)	
Unemployed/student/retirement	275 (29.5)	65 (28.4)	60 (30.8)	47 (27.6)	11 (20.8)	47 (32.9)	45 (31.7)	
Health literacy^c^								<0.001
Adequate	850 (92.8)	228 (99.5)	188 (96.4)	169 (99.4)	39 (78.0)	108 (78.8)	118 (87.4)	
Low	66 (7.2)	1 (0.4)	7 (3.6)	1 (0.6)	11 (22.0)	29 (21.2)	17 (12.6)	
Missing	16 (1.7)	0 (0.0)	0 (0.0)	0 (0.0)	3 (5.7)	6 (4.2)	7 (4.9)	
Difficulty with Dutch language^c^								<0.001
No	463 (67.7)	NA	147 (75.8)	149 (88.2)	11 (22.0)	75 (55.1)	81 (60.0)	
Yes	221 (32.3)	NA	47 (24.2)	20 (11.8)	39 (78.0)	61 (44.9)	54 (40.0)	
Missing	19 (2.0)	NA	1 (0.5)	1 (0.6)	3 (5.7)	7 (4.9)	7 (4.9)	
Cultural orientation^c^								
Less integrated	95 (14.2)	NA	25 (13.0)	20 (11.9)	8 (16.7)	24 (18.2)	18 (14.1)	
More integrated	574 (85.8)	NA	168 (87.0)	148 (88.1)	40 (83.3)	108 (81.8)	110 (85.9)	
Missing	34 (4.8)	NA	2 (1.0)	2 (1.2)	5 (9.4)	11 (7.7)	14 (9.9)	
Health factors								
Smoking^e^								<0.001
Non smoker	473 (50.9)	78 (34.1)	114 (58.5)	82 (48.2)	37 (69.8)	61 (43.0)	101 (72.1)	
Former smoker	302 (32.5)	120 (52.4)	44 (22.6)	60 (35.3)	13 (24.5)	42 (29.6)	23 (16.4)	
Current smoker	154 (16.6)	31 (13.5)	37 (19.0)	28 (16.5)	3 (5.7)	39 (27.5)	16 (11.4)	
Missing	3 (0.3)	0 (0.0)	0 (0.0)	0 (0.0)	0 (0.0)	1 (0.7)	2 (1.4)	
BMI^e^								
Normal	315 (34.3)	130 (57.0)	65 (33.7)	39 (23.2)	10 (19.2)	36 (26.1)	35 (25.0)	
Overweight	385 (41.9)	71 (31.1)	88 (45.6)	85 (50.6)	24 (46.2)	55 (39.9)	62 (44.3)	
Obese	219 (23.8)	27 (11.8)	40 (20.7)	44 (26.2)	18 (34.6)	47 (34.1)	43 (30.7)	
Missing	13 (1.4)	1 (0.4)	2 (1.0)	2 (1.2)	1 (1.9)	5 (3.5)	2 (1.4)	
Comorbidity^e^								
No	503 (54.2)	124 (54.4)	97 (49.7)	76 (44.7)	20 (37.7)	94 (66.7)	92 (65.2)	<0.001
1 or more	425 (45.8)	104 (45.6)	98 (50.3)	94 (55.3)	33 (62.3)	47 (33.3)	49 (34.8)	
Missing	4 (0.4)	1 (0.4)	0 (0.0)	0 (0.0)	0 (0.0)	2 (1.4)	1 (0.7)	
CVD^e^								0.413
No	880 (94.8)	215 (93.9)	183 (93.8)	160 (94.1)	49 (94.2)	135 (95.1)	138 (98.6)	
Yes	48 (5.2)	14 (6.1)	12 (6.2)	10 (5.9)	3 (5.8)	7 (4.9)	2 (1.4)	
Missing	4 (0.4)	0 (0.0)	0 (0.0)	0 (0.0)	1 (1.9)	1 (0.7)	2 (1.4)	
Asthma/COPD^e^								0.118
No	887 (95.2)	213 (93.0)	181 (92.8)	165 (97.1)	52 (98.1)	138 (96.5)	138 (97.2)	
Yes	45 (4.8)	16 (7.0)	14 (7.2)	5 (2.9)	1 (1.9)	5 (3.5)	4 (2.8)	
Missing	0 (0.0)	0 (0.0)	0 (0.0)	0 (0.0)	0 (0.0)	0 (0.0)	0 (0.0)	
Diabetes^e^								<0.001
No	819 (88.2)	211 (92.5)	153 (78.5)	153 (90.5)	44 (83.0)	131 (92.3)	127 (89.4)	
Yes	110 (11.8)	17 (7.5)	42 (21.5)	16 (9.5)	9 (17.0)	11 (7.7)	15 (10.6)	
Missing	3 (0.3)	1 (0.4)	0 (0.0)	1 (0.6)	0 (0.0)	1 (0.7)	0 (0.0)	
Hypertension^e^								<0.001
No	556 (59.7)	135 (59.0)	111 (56.9)	81 (47.6)	24 (45.3)	105 (73.4)	100 (70.4)	
Yes	376 (40.3)	94 (41.0)	84 (43.1)	89 (52.4)	29 (54.7)	38 (26.6)	42 (29.6)	
Missing	0 (0.0)	0 (0.0)	0 (0.0)	0 (0.0)	0 (0.0)	0 (0.0)	0 (0.0)	

Abbreviations: HELIUS, Healthy life in an urban setting; COVID-19, Coronavirus disease 2019; IQR, interquartile range; SARS-CoV-2, severe acute respiratory syndrome coronavirus; COPD, chronic obstructive pulmonary disease; BMI, body mass index; CVD, cardiovascular disease; DM, diabetes mellitus; GP, general practitioner; ICU, intensive care unit; NA, not applicable.

a Pearson’s χ2 or Kruskal-Wallis test, as appropriate.

b Age at the infection date.

c Measured at HELIUS baseline (2011–2015).

d Measured during the third COVID-19 substudy visit (May–November 2022).

e Measured at HELIUS follow-up (2019–2021).

**Table 2.** SARS-CoV-2 related characteristics of the HELIUS participants reporting a past SARS-CoV-2 infection, overall and per ethnic group, Amsterdam, the Netherlands, May 17, 2022 to November 21, 2022 (*n* = 932) Table 2. long description.

Characteristic	Total (*n* = 932)	Dutch (*n* = 229)	South-Asian Surinamese (*n* = 195)	African Surinamese (*n* = 170)	Ghanaian (*n* = 53)	Turkish (*n* = 143)	Moroccan (*n* = 142)	*P* value^a^
	*n* (%)	*n* (%)	*n* (%)	*n* (%)	*n* (%)	*n* (%)	*n* (%)	
SARS-CoV-2 related factors								
Number of past infections^b^								<0.001
1	724 (77.7)	195 (85.2)	164 (84.1)	134 (78.8)	42 (79.2)	95 (66.4)	94 (66.2)	
2	191 (20.5)	31 (13.5)	29 (14.9)	31(18.2)	11 (20.8)	46 (32.2)	43 (30.3)	
3	15 (1.6)	3 (1.3)	2 (1.0)	4 (2.4)	0 (0.0)	2 (1.4)	4 (2.8)	
4	2 (0.2)	0 (0.0)	0 (0.0)	1 (0.6)	0 (0.0)	0 (0.0)	1 (0.7)	
Time (months) between date of (most severe) infection and date of the third substudy visit, median (IQR)^b^	8.9 (5.1, 20.4)	5.4 (3.7, 11.6)	9.1 (5.4, 20.2)	9.1 (4.9, 20.3)	17.5 (7.8, 23.1)	15.6 (7.1, 20.9)	15.4 (7.1, 21.8)	<0.001
Most severe infection^b^								<0.001
Participants with one infection	724 (77.7)	195 (85.2)	164 (84.1)	134 (78.8)	42 (79.2)	95 (66.4)	94 (66.2)	
1^st^ infection	152 (16.3)	18 (7.9)	23 (11.8)	26 (15.3)	7 (13.2)	41 (28.7)	37 (26.1)	
2^nd^ infection	55 (5.9)	15 (6.6)	8 (4.1)	10 (5.9)	4 (7.5)	7 (4.9)	11 (7.7)	
3^rd^ infection	1 (0.1)	1 (0.4)	0 (0.0)	0 (0.0)	0 (0.0)	0 (0.0)	0 (0.0)	
SARS-CoV-2 vaccination status and calendar period of infection reflecting the dominant variant circulating in the Netherlands^b^ ^,^^c^ ^,^^d^								<0.001
Infected in the Pre-Alpha period	307 (33.0)	41 (17.9)	63 (32.3)	58 (34.1)	25 (47.2)	56 (39.2)	64 (45.4)	
Unvaccinated and infected in the Alpha-Delta period	44 (4.7)	5 (2.2)	5 (2.6)	11 (6.5)	1 (1.9)	7 (4.9)	15 (10.6)	
Vaccinated and infected in the Alpha-Delta period	148 (15.9)	33 (14.4)	39 (20.0)	24 (14.1)	10 (18.9)	25 (17.5)	17 (12.1)	
Unvaccinated and infected in the Omicron period	51 (5.5)	6 (2.6)	10 (5.1)	17 (10.0)	1 (1.9)	6 (4.2)	11 (7.8)	
Vaccinated and infected in the Omicron	381 (40.9)	144 (62.9)	78 (40.0)	60 (35.3)	16 (30.2)	49 (34.3)	34 (24.1)	
Missing	1 (0.1)	0 (0.0)	0 (0.0)	0 (0.0)	0 (0.0)	0 (0.0)	1 (0.7)	
Hospital admission due to COVID-19^b^ ^,^^e^								0.539
No	861 (96.4)	219 (97.8)	171 (94.5)	179 (94.7)	47 (100.0)	136 (97.8)	128 (94.8)	
Hospital admission	23 (2.6)	4 (1.8)	7 (3.7)	6 (3.8)	0 (0.0)	1 (0.7)	5 (3.7)	
ICU admission	9 (1.0)	1 (0.4)	3 (1 .6)	1 (0.6)	0 (0.0)	2 (1.4)	2 (1.5)	
Missing	39 (4.2)	5 (2.2)	6 (3.1)	11 (6.5)	6 (11.3)	4 (2.8)	7 (4.9)	
Sick leave due to COVID-19-related symptoms in the 28 days following infection^b^ ^,^^c^								
No	226 (24.2)	80 (34.9)	36 (18.5)	35 (20.6)	17 (32.1)	29 (20.3)	29 (20.4)	0.001
1–9 days	206 (22.1)	56 (24.5)	44 (22.6)	41 (24.1)	11 (20.8)	31 (21.7)	23 (16.2)	
10–28 days	225 (24.1)	28 (12.2)	55 (28.2)	47 (27.6)	14 (26.4)	36 (25.2)	45 (31.7)	
*Not working*	275 (29.5)	65 (28.4)	60 (30.8)	47 (27.6)	11 (20.8)	47 (32.9)	45 (31.7)	
SARS-CoV–2 antibody test result at previous substudy visit^e^ ^,^^f^								<0.001
Negative at the second COVID-19 substudy visit	601 (68.4)	179 (82.1)	134 (73.6)	109 (66.9)	20 (43.5)	79 (59.0)	80 (59.7)	
Positive at the first and/or second COVID-19 substudy visit	276 (31.6)	39 (17.9)	48 (26.4)	54 (33.1)	26 (56.5)	55 (41.0)	54 (40.3)	
Missing	55 (6.2)	11 (4.8)	13 (6.7)	7 (4.1)	7 (13.2)	9 (6.3)	8 (5.6)	
Having at least one COVID-19-related symptom in the first week since infection^b^								0.045
No	46 (4.9)	9 (3.9)	7 (3.6)	11 (6.5)	7 (13.2)	4 (2.8)	8 (5.6)	
Yes	886 (95.1)	220 (96.1)	188 (96.4)	159 (93.5)	46 (86.8)	139 (97.2)	134 (94.4)	
Number of COVID-19-related symptoms in the first week since infection, median (IQR)^g^	7.0 (4.0, 9.0)	5.0 (3.0, 7.0)	7.0 (5.0, 10.0)	6.0 (4.0, 9.0)	6.0 (4.3, 8.0)	7.0 (5.0, 10.0)	7.0 (5.0, 10.0)	<0.001

Abbreviations: HELIUS, healthy life in an urban setting; COVID-19, coronavirus disease 2019; IQR, interquartile range; SARS-CoV-2, severe acute respiratory syndrome coronavirus; ICU, intensive care unit; NA, not applicable.

a Pearson’s χ2 or Kruskal-Wallis test, as appropriate.

b Measured during the third COVID-19 substudy visit (May–November 2022).

c SARS-CoV-2 vaccination status was determined by the question ‘Which primary vaccinations have you received?’. Incomplete: received one dose of a vaccine other than Janssen, with or without subsequent infection; complete: received two doses of Pfizer, Moderna or AstraZeneca, ≥1 dose of Janssen, or had a past infection and subsequently received ≥1 dose of any vaccine (based on the guidelines of the Dutch government, reference: National Institute for Public Health and the Environment. COVID-19-vaccinatie uitvoeringsrichtlijn – version 4 December 2021. 2021. Available from: https://lci.rivm.nl/richtlijnen/covid-19-vaccinatie. Accessed 29 April 2025). Only two participants were vaccinated incompletely and were therefore grouped with the complete group under the category ‘vaccinated’.

d Calendar period of infection is categorized based on the prevalent SARS-CoV-2 variant circulating in the Netherlands.

e Having a positive SARS-CoV-2 antibody test result due to prior infection was defined as having a positive antibody test result from the first and/or the second COVID-19 substudy visits (visit 1: June–October 2020 or visit 2: November 2020–June 2021, before the COVID-19 vaccination was fully implemented in the Netherlands). Having a negative SARS-CoV-2 antibody test result due to prior infection was defined as having a negative antibody test result from the second COVID-19 substudy visit.

f One participant with an equivocal test result from the second COVID-19 substudy, and 54 participants with a negative test result from the first COVID-19 substudy visit who did not participate the second COVID-19 substudy visit are categorized as missing.

g Calculated among participants having at least one COVID-19-related symptom in the first week since infection.

### Prevalence of COVID-19-related symptoms and PCC over time

The median time between infection and the third substudy visit was 8.9 months (IQR = 5.1–20.4). During reconstructed follow-up, 46 (5%) participants did not report any symptoms, while 271/851 (32%) and 142/398 (36%) reported at least one COVID-19-related symptom at month 3 and 12, respectively. The most common COVID-19-related symptoms at both month 3 and month 12 were fatigue, impaired concentration, and memory problems for all ethnic groups except for those of Ghanaian and Dutch origin. For participants of Ghanaian origin, the most common symptoms were muscle pain, hair loss, and fatigue at month 3, and fatigue, muscle pain, and hair loss at month 12; for participants of Dutch origin the most common symptoms were fatigue, loss of taste or smell, and concentration problems at month 3, and fatigue, loss of taste or smell, and shortness of breath at month 12 (Supplementary Figure S2).

**Figure 1.**
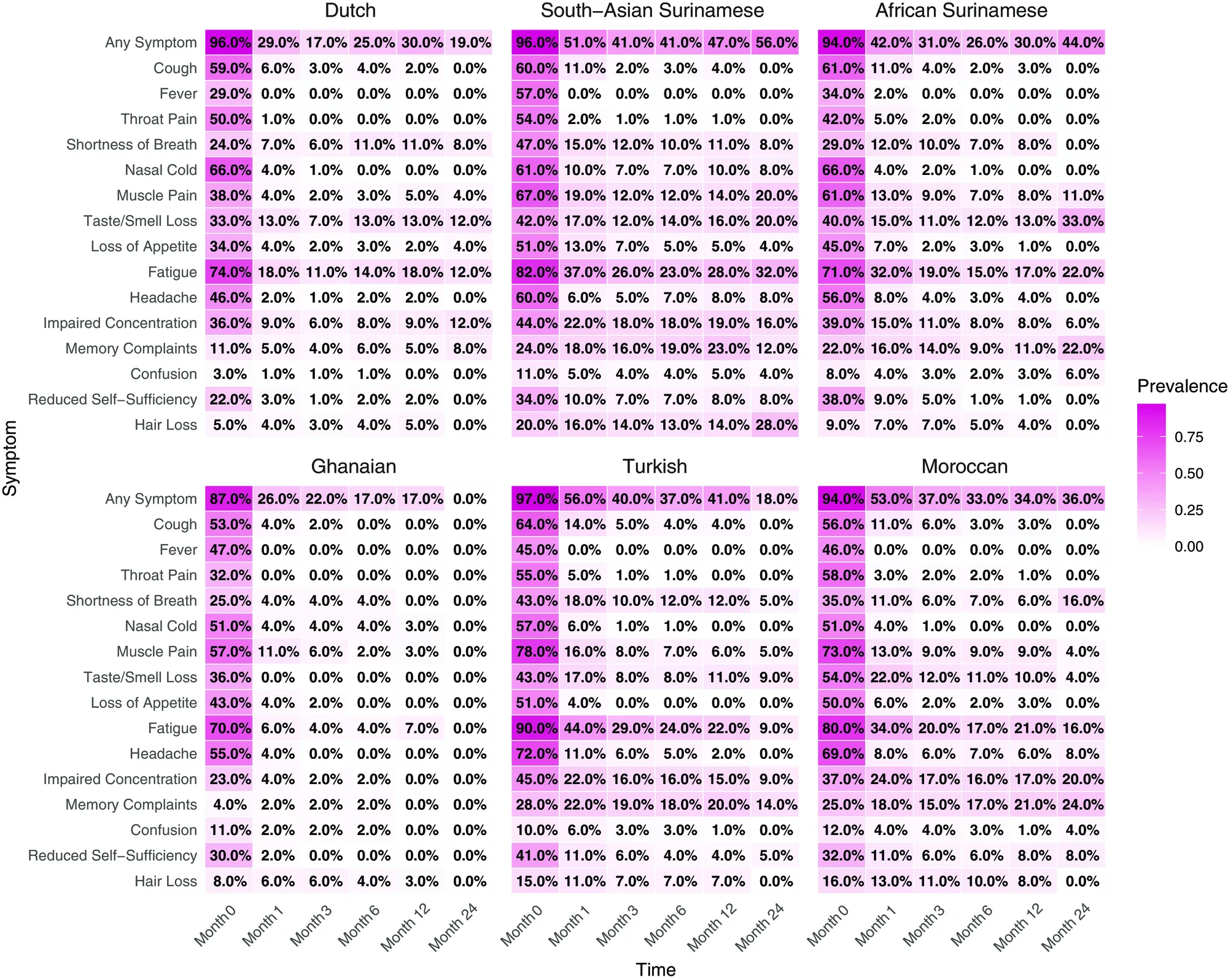
Heatmap of the prevalence of COVID-19-related symptoms during follow-up (i.e, months since infection) adjusted for sex and age, per ethnic group, the HELIUS COVID-19 substudy, May–November 2022, Amsterdam, the Netherlands (*n* = 932). The reconstructed follow-up involved taking the time of infection (as month 0) and reconstructing timepoints until the date when the questionnaire was filled out (i.e., months 1, 3, 6, 12, 24). For instance, if a participant filled out a questionnaire at month 6 of the reconstructed timeline, we could infer the presence of COVID-19-related symptoms at months 1, 3, and 6 but we could not infer the presence of COVID-19-related symptoms at months 12 or 24. Figure 1. long description.

The prevalence of any and specific COVID-19-related symptoms during follow-up, adjusted for age and sex, is shown in Figure 1 per ethnic group (Supplementary Table S2). At month 3, the adjusted prevalence of PCC varied from 17% (95%CI: 13%–24%) in the Dutch-origin group to 41% (95%CI: 34%–48%) in the South-Asian Surinamese group. Prevalence was significantly higher among participants of South-Asian Surinamese, Turkish and Moroccan origin compared to those of Dutch origin (all *P* < 0.001). At month 12, the adjusted prevalence was lowest at 17% in the Ghanaian group (95%CI: 7%–35%) and highest at 47% in the South Asian Surinamese group (95%CI: 36%–58%) (Supplementary Table S2); however, overall differences were not significant (*P* = 0.261, 0.338, 0.387, 0.750, and 0.654 for participants of South-Asian Surinamese, African Surinamese, Ghanaian, Turkish, and Moroccan origin, respectively).

### Determinants of PCC

**Figure 2.**
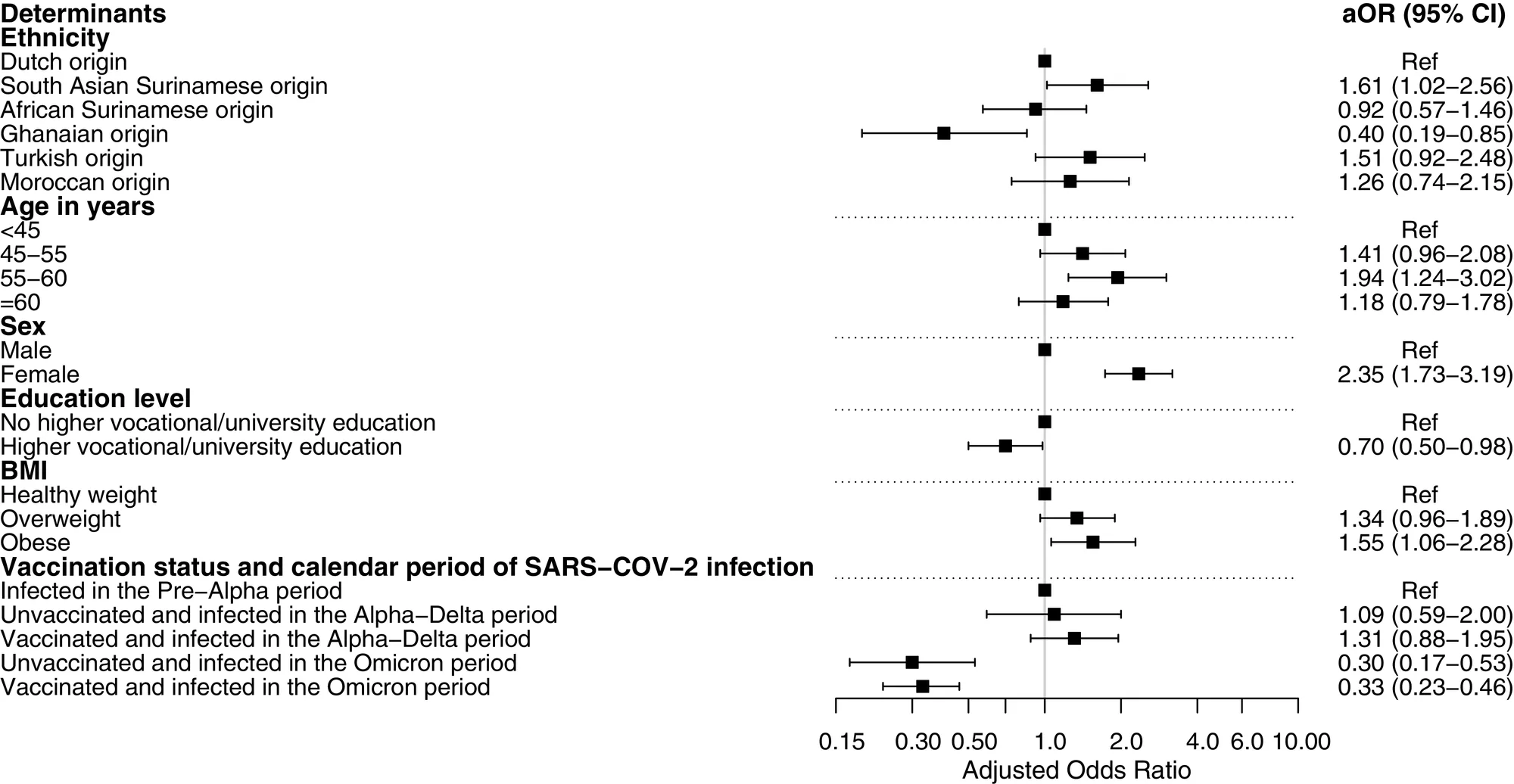
Forest plot of adjusted odds ratios of determinants of PCC from multivariable model, the HELIUS COVID-19 substudy, May–November 2022, Amsterdam, the Netherlands. Multivariable model (*n* = 819, i.e. participants reporting past infection acquired at least 3 months ago). Abbreviations: PCC, post-COVID-19 condition; SARS-CoV-2, severe acute respiratory syndrome coronavirus; BMI, body mass index; aOR, adjusted odds ratio; CI, confidence interval. The multivariable model was obtained by forcing ethnicity, age, and sex variables into the model and removing the other covariates with a *P* ≥ 0.05 in a backward stepwise fashion using a set of potential determinants. Participants who have missing covariate values are excluded from the analysis. Figure 2. long description.

The univariable analysis of determinants for PCC is presented in Supplementary Table S3. In the multivariable model, participants of South-Asian Surinamese origin had higher odds, and those of Ghanaian origin had lower odds of reporting PCC symptoms during follow-up than participants of Dutch origin (overall *P* = 0.002) (Figure 2 and Supplementary Table S4). The higher odds of PCC for the non-Dutch ethnic groups were attenuated compared to the univariable analyses. Additionally, the odds of PCC over time were increased for females (overall *P* < 0.001), participants aged 55–59 years (overall *P* = 0.023), and participants who were obese (overall *P* = 0.063). Participants with a higher vocational or university degree (overall *P* = 0.038), and both vaccinated and unvaccinated participants infected during the Omicron period (overall *P* < 0.001) had lower odds of PCC over time. There were no significant interactions between ethnicity and the other variables in the model.

In post hoc analysis, adding sick leave within the first 4 weeks after infection to the multivariable model (while including only employed participants without previous leave) attenuated the difference between the South Asian Surinamese group (aOR: 0.96, 95% CI: 0.54–1.71) compared to the Dutch group (Supplementary Table S4). Migration-related variables were not associated with probability of PCC over time (Supplementary Table S3–S4).

### Sensitivity analysis

When additionally including individuals who did not report infection despite a positive SARS-CoV-2 antibody test (*n* = 60), the prevalence of PCC across ethnic groups was somewhat lower in some groups but patterns over time remained consistent with our main findings (Supplementary Table S5). The association between ethnicity and PCC was similar to the main analysis, except that odds of PCC during follow-up among participants of the South Asian Surinamese origin, compared to those of Dutch origin, were attenuated (aOR: 1.42, 95% CI: 0.92–2.18), and the participants of the Ghanaian group had lower odds of PCC during follow-up compared to participants of Dutch origin in the multivariable (aOR: 0.16; 95% CI: 0.08–0.34) (Supplemental Table S6).

## Discussion

In this population-based, multi-ethnic cohort study, the prevalence of PCC remained higher over time since infection in participants of South-Asian Surinamese, Turkish, and Moroccan origin, compared to those of Dutch origin. The most common COVID-19-related symptoms at both month 3 and month 12 were fatigue, impaired concentration, and memory problems for all ethnic groups except for those of Ghanaian and Dutch origin. In multivariable analysis, certain risk factors of PCC emerged, including being older, female, obese, not having a higher vocational or university degree and acquiring SARS-CoV-2 during the pre-omicron period, while the associations of these determinants with PCC did not differ across ethnic groups.

Our findings were in line with registry-based studies from Denmark and Sweden, in which the risk of long COVID was increased among migrants from Africa [9] and the Middle East [8, 9]. There has been another study in the Netherlands conducted in specifically hospitalized individuals where the risk of PCC was elevated in participants of South-Asian Surinamese, African Surinamese, Turkish and Moroccan background compared to those of Dutch origin [7]. Even after a reconstructed follow-up of 24 months after infection, we observed that between-ethnic differences remained present while adjusting for age and sex in a population-based setting. Nevertheless, it should be mentioned that there were other determinants in the multivariable model, such as sex and calendar period of acquiring SARS-CoV-2 infection, with stronger effect sizes than ethnicity.

Interestingly, the ethnic differences in PCC risk mirror those of SARS-CoV-2 infection and disease severity in Amsterdam, with the exception of participants of Ghanaian origin [5]. In a previous analysis using data from the first HELIUS substudy, we observed that 51% of participants in the Ghanaian-origin group who had SARS-CoV-2 antibodies did not present with any symptoms, while this proportion was much lower in the other ethnic groups [13]. We also observed a much lower proportion of symptomatic infection in the Ghanaian group, at 87%, in the present study among those who reported an infection. These findings are in the context of higher rates of infection in those with Ghanaian origin compared to other ethnic groups [5, 13]. A possible explanation for the lower burden of COVID-19 symptoms and PCC could be differences in host responses related to differences in immunological genetic factors [21, 22], and warrants further investigation. It could also be that certain ethnic groups may report having fewer symptoms or have different susceptibilities to symptoms regardless of having PCC [8]. In an informal analysis of HELIUS substudy participants who did not report a past SARS-CoV-2 infection, the prevalence of having any symptom 2 weeks prior to the study visit was not different between ethnic groups after adjusting for age, sex, and season (data not shown).

One reason why the prevalence of PCC could be higher in some migrant origin groups is that those with a migration background have a higher probability of presenting with severe COVID-19 disease [4], and severe COVID-19 could increase the risk of PCC [7, 23–26]. As such, disease severity becomes an explanatory variable in the association between ethnicity and PCC. As the number of individuals hospitalized or admitted to intensive care was too small to include in the analysis, we used sick leave in the first 4 weeks after infection as a proxy for disease severity and included it in our multivariable model. In this adjustment, we observed that differences in PCC between ethnic groups became attenuated, similar to another study [9]. Disease severity might partly explain ethnic differences in the prevalence of PCC.

A wide body of research has already highlighted the association of older age, being female, and not having a higher vocational or university degree on PCC [25], while our study demonstrates that these same associations do not differ across ethnic groups. Others have also shown that PCC was less common among participants infected during the Omicron period compared to pre-Omicron periods [9, 27], while again this association was no different across ethnic groups in our study. However, assessing the effect of the Omicron variant is confounded by increased vaccination rates during the Omicron-dominant period [28]. Despite careful attention to separating vaccination and period in our multivariable model, we did not find any additional association between being vaccinated and PCC within the period of dominant variants of interest. Nevertheless, we were unable to identify whether infections occurred prior to or after vaccination at the individual level, making it difficult to fully infer the temporality of vaccination on PCC [29]. Most data point towards a protective effect on PCC from pre-infection vaccination [30].

The majority of individuals with PCC do not experience resolution and generally have worse health outcomes compared to those without PCC [31, 32]. Considering the higher PCC prevalence among several groups with a migration background, our findings underscore the need for sufficient access to post-COVID care in these populations. As ethnic minorities might exhibit different manifestations of PCC-related symptoms compared to majority populations [7, 8], clinicians might not recognize PCC [33, 34], and thus may not refer them to specialized services for PCC-related care [8]. Moreover, care for PCC typically involves general practitioners, multi-disciplinary specialist care, and post-COVID clinics [35, 36]. It is unclear whether these services are fully adapted to populations with a migration background, especially as their health-seeking behaviours and care utilization patterns can be different [37, 38]. A pre-pandemic study from the Netherlands reported lower utilization of specialized health care services for migrant groups, potentially because of limited access [39]. Even though all residents of the Netherlands are obliged to have standard health insurance, which provides access to primary and secondary health care, financial barriers might hinder equitable access to PCC-related care (e.g., the cost of secondary specialized PCC care is only covered for healthcare expenditure over the annual deductible) [40]. Future research should focus on access to specialized PCC care across ethnic groups.

This population-based cohort is strengthened by a well-designed structure to address clinical questions during the COVID-19 pandemic, extensive data on the presence of COVID-19-related symptoms since SARS-CoV-2 infection, and few missing data. Our study encompasses the largest ethnic groups in the Netherlands; therefore, our findings are likely to be representative for these ethnic minority groups in urban regions in Western Europe. However, generalizability across ethnic groups living in rural areas, undocumented migrants, asylum seekers, and refugees is limited. Nevertheless, our study has some limitations. First, we did not have data on the presence of symptoms prior to SARS-CoV-2 infection, which makes it difficult to exclude symptoms unlikely to be the result of COVID-19 [41]. Consequently, the prevalence of PCC could be overestimated [42, 43], and this overestimation may have differed across ethnic groups. However, after adjusting for pre-infection health-related factors (i.e. BMI), the differences in PCC between ethnic groups remained. In addition, we did not have data on severity of symptoms. Examining the relationship between PCC and sick leave across ethnic groups might be informative and will be explored in our future studies. Second, infection status, symptoms, and duration of symptoms were collected retrospectively and thus could be subject to recall or reporting bias. Third, although participants were tested for past SARS-CoV-2 and were informed about the test results at all substudy visits, some individuals may have ignored or not recalled their asymptomatic infection, and hence did not report being infected at the third study visit. We did address this potential selection bias through our sensitivity analyses, additionally including those who did not report infection despite their positive SARS-CoV-2 antibody result. The results were mostly unchanged, except for lower risk of PCC in participants of Ghanaian and South-Asian Surinamese origin. Lastly, due to reconstructing time intervals from cross-sectional data, participants with more recent infections had shorter follow-up times. Estimates at months 12 and 24 would then more often represent those infected when the alpha-delta and pre-alpha variants were dominant, respectively. Continued follow-up for those with more recent infections is needed in further studies.

In conclusion, there is a higher prevalence of PCC following infection among individuals of South-Asian Surinamese, Turkish and Moroccan origin, and those of Ghanaian origin were less likely to have PCC compared to those of Dutch origin. Nevertheless, these results are in the context of stronger associations with other determinants, such as sex and calendar period of acquiring SARS-CoV-2 infection. Since the symptoms involved in PCC could be different across ethnic groups, clinicians need to be aware of the potential for ethnic-specific manifestations of PCC and the burden of PCC.

## Supporting information

10.1017/S095026882610199X.sm001 Spronk et al. supplementary material Spronk et al. supplementary material

## Data Availability

The HELIUS data are owned by the Amsterdam University Medical Center, location AMC in Amsterdam, The Netherlands. Any researcher can request the data by submitting a proposal to the HELIUS Executive Board as outlined at http://www.heliusstudy.nl/en/researchers/collaboration, by email: heliuscoordinator@amsterdamumc.nl. The HELIUS Executive Board will check proposals for compatibility with the general objectives, ethical approvals and informed consent forms of the HELIUS study. There are no other restrictions to obtaining the data, and all data requests will be processed in the same manner.

## Supplementary material

The supplementary material for this article can be found at http://doi.org/10.1017/S095026882610199X.

## Long descriptions

### Table 1. Long description

A table with 9 columns and multiple rows categorized into Sociodemographic factors and Health factors. The columns represent the Total population (n = 932) and six ethnic groups: Dutch (n = 229), South-Asian Surinamese (n = 195), African Surinamese (n = 170), Ghanaian (n = 53), Turkish (n = 143), and Moroccan (n = 142), followed by a P value column.

Key Sociodemographic Data:

* Age: The median age is 55 years. The Dutch group is the oldest (median 59) and the Moroccan group is the youngest (median 50).

* Sex: 60.6% of the total are female, with the highest proportion in the African Surinamese group (68.2%).

* Education: 66.8% of Dutch participants have higher vocational or university education, compared to only 12.0% of Ghanaian participants.

* Migration: Most participants are 1st generation (79.1% overall).

* Health Literacy: Generally high (92.8% adequate), but lower in Ghanaian (78.0%) and Turkish (78.8%) groups.

Key Health Factors:

* Smoking: 50.9% are non-smokers. The highest current smoking rate is in the Turkish group (27.5%).

* B M I: 23.8% are obese. Obesity is highest in Ghanaian (34.6%) and Turkish (34.1%) groups, and lowest in the Dutch group (11.8%).

* Comorbidity: 45.8% have 1 or more comorbidities, peaking in the Ghanaian group (62.3%).

* Hypertension: 40.3% overall, with the highest prevalence in Ghanaian (54.7%) and African Surinamese (52.4%) groups.

* Diabetes: 11.8% overall, with a significantly higher prevalence in the South-Asian Surinamese group (21.5%).

Navigate back to Table 1..

### Table 2. Long description

The table presents data for 932 participants across eight columns: Characteristic, Total, Dutch, South-Asian Surinamese, African Surinamese, Ghanaian, Turkish, Moroccan, and P value.

Key data points include:

* Number of past infections: 77.7 percent of the total had one infection, while 20.5 percent had two. Turkish and Moroccan groups had higher rates of two infections at 32.2 percent and 30.3 percent respectively (P < 0.001).

* Time between infection and visit: The median was 8.9 months total, but significantly lower for the Dutch group at 5.4 months compared to 17.5 months for the Ghanaian group.

* Vaccination and calendar period of infection reflecting the dominant variant circulating in the Netherlands: 40.9 percent of the total were vaccinated and infected during the Omicron period. This was highest in the Dutch group at 62.9 percent and lowest in the Moroccan group at 24.1 percent.

* Hospitalization: 2.6 percent of the total were admitted to the hospital, and 1.0 percentof the total were admitted to the I C U. Differences between groups were not statistically significant (P = 0.539).

* Sick leave: 24.1 percent of the total took 10 to 28 days of sick leave. The Moroccan group had the highest rate in this category at 31.7 percent, while the Dutch group had the lowest at 12.2 percent.

* Symptoms: 95.1 percent of participants reported at least one symptom in the first week. The median number of symptoms was 7.0 overall, with the Dutch group reporting fewer (median 5.0) compared to South-Asian Surinamese, Turkish, and Moroccan groups (median 7.0).

Navigate back to Table 2..

### Figure 1. Long description

The heatmap displays prevalence percentages for 16 symptoms across six ethnic groups. The y-axis lists symptoms: Any Symptom, Cough, Fever, Throat Pain, Shortness of Breath, Nasal Cold, Muscle Pain, Taste/Smell Loss, Loss of Appetite, Fatigue, Headache, Impaired Concentration, Memory Complaints, Confusion, Reduced Self-Sufficiency, and Hair Loss. The x-axis represents time points: Month 0, 1, 3, 6, 12, and 24. A color scale on the right indicates prevalence from 0.00 (white) to 0.75 or higher (dark pink).

* Dutch: Any Symptom prevalence is 96.0% at Month 0, and 19.0% at Month 24. Fatigue remains relatively high compared to other specific symptoms, ending at 12.0%.

* South-Asian Surinamese: Shows higher fatigue prevalence at 12 and 24 months compared to other groups. Any Symptom starts at 96.0% and remains at 56.0% at Month 24.

* African Surinamese: Any Symptom starts at 94.0% and ends at 44.0%. Taste/Smell Loss is notably high at Month 24 (33.0%).

* Ghanaian: Any Symptom prevalence is 87.0% at Month 0, and 0.0% at Month 24.

* Turkish: Any Symptom starts at 97.0% and ends at 18.0%. .

* Moroccan: Any Symptom starts at 94.0% and remains at 36.0% at Month 24.

Across all groups, fatigue and cognitive symptoms like impaired concentration show more persistence.

Navigate back to Figure 1..

### Figure 2. Long description

A forest plot with a vertical reference line at 1.0 representing the null effect. The x-axis at the bottom measures Adjusted Odds Ratio on a logarithmic scale from 0.15 to 10.00.

* Ethnicity: Dutch origin is the reference. South Asian Surinamese origin shows an a O R of 1.61 (95% C I 1.02 to 2.56). African Surinamese origin is 0.92 (0.57 to 1.46). Ghanaian origin is 0.40 (0.19 to 0.85). Turkish origin is 1.51 (0.92 to 2.48). Moroccan origin is 1.26 (0.74 to 2.15).

* Age in years: Less than 45 is the reference. 45 to 55 is 1.41 (0.96 to 2.08). 55 to 60 is 1.94 (1.24 to 3.02). 60 or older is 1.18 (0.79 to 1.78).

* Sex: Male is the reference. Female is 2.35 (1.73 to 3.19).

* Education level: No higher vocational or university education is the reference. Higher vocational or university education is 0.70 (0.50 to 0.98).

* B M I: Healthy weight is the reference. Overweight is 1.34 (0.96 to 1.89). Obese is 1.55 (1.06 to 2.28).

* Vaccination status and calendar period of S A R S-C o V-2 infection: Infected in the Pre-Alpha period is the reference. Unvaccinated and infected in the Alpha-Delta period is 1.09 (0.59 to 2.00). Vaccinated and infected in the Alpha-Delta period is 1.31 (0.88 to 1.95). Unvaccinated and infected in the Omicron period is 0.30 (0.17 to 0.53). Vaccinated and infected in the Omicron period is 0.33 (0.23 to 0.46).

Navigate back to Figure 2..
