# Supplementary material for: Presence of post-COVID-19 condition in six ethnic groups in Amsterdam, the Netherlands: a retrospectively reconstructed time interval approach
Source: Epidemiol Infect. 2026 Oct 14;154:e121. doi: 10.1017/S095026882610199X (PMC13749104; doi:10.1017/S095026882610199X)
Supplement: Supplementary file 1 [file S095026882610199Xsup001.docx]

**Supplementary materials to:**

**Presence of post COVID-19 condition in six ethnic groups in Amsterdam, the Netherlands: a retrospectively reconstructed time interval approach**

Merve Spronk ^a b c^, Anders Boyd ^a b c d^, Sophie Campman ^a b c^, Vita Jongen ^a c d^, Henrike Galenkamp ^g h^, Felix P. Chilunga ^g h^, Charles Agyemang ^g i^, Janke Schinkel ^j^, Elke Wynberg ^k^, Karien Stronks ^g h^, Maria Prins ^a b c^

**Affiliations**

^a^ Department of Infectious Diseases, Public Health Service of Amsterdam, Amsterdam, the Netherlands

^b^ Amsterdam UMC location University of Amsterdam, Infectious Diseases, Meibergdreef 9, Amsterdam, the Netherlands

^c^ Amsterdam Institute for Infection and Immunity, Infectious Diseases, Amsterdam, the Netherlands

^d^ Stichting hiv monitoring, Amsterdam, the Netherlands

^e^ Department of Health Sciences, Faculty of Science, Vrije Universiteit, Amsterdam, the Netherlands

^f^ Amsterdam Public Health Research Institute, the Netherlands

^g^ Amsterdam UMC location University of Amsterdam, Department of Public and Occupational Health, Amsterdam, the Netherlands

^h^ Amsterdam Public Health, Health Behaviours and Chronic Diseases, Amsterdam, the Netherlands

^i^ Department of Medicine, Division of Endocrinology, Diabetes and Metabolism, Johns Hopkins University School of Medicine, Baltimore, MD, USA

^j^ Amsterdam UMC location University of Amsterdam, Department of Medical Microbiology and Infection Prevention, Meibergdreef 15, Amsterdam, the Netherlands

^k^ Mahidol-Oxford Tropical Medicine Research Unit (MORU), Faculty of Tropical Medicine, Mahidol University, Bangkok, Thailand

TABLE OF CONTENT

[Supplementary Methods	3](#_Toc229055931)

[Assessing COVID-19 related symptoms	3](#_Toc229055932)

[Supplementary Figures	6](#_Toc229055933)

[Figure S1. Flowchart of the inclusion of HELIUS participants in the third visit of the COVID-19 substudy, Amsterdam, the Netherlands, May 17 - November 21, 2022.	6](#_Toc229055934)

[Figure S2. Radar plots presenting the proportion of each COVID-19 related symptom by ethnic group at month 3 and 12 by ethnicity, the HELIUS COVID-19 substudy, May-November 2022, Amsterdam, the Netherlands.	8](#_Toc229055935)

[Supplementary Tables	9](#_Toc229055936)

[Table S1. Characteristics of the HELIUS third COVID-19 participants, by inclusion in the present study, the HELIUS COVID-19 substudy, May-November 2022, Amsterdam, the Netherlands (n=1505).	9](#_Toc229055937)

[Table S2. Adjusted prevalence estimates of having any COVID-19 related symptom during follow-up by ethnicity, the HELIUS COVID-19 substudy, May-November 2022, Amsterdam, the Netherlands. Main analysis among 932 participants reporting a past SARS-CoV-2 infection.	12](#_Toc229055938)

[Table S3. Determinants of PCC among HELIUS participants - Univariable analysis, the HELIUS COVID-19 substudy, May-November 2022, Amsterdam, the Netherlands.	13](#_Toc229055939)

[Table S4. Determinants of PCC among HELIUS participants - Multivariable analysis, the HELIUS COVID-19 substudy, May-November 2022, Amsterdam, the Netherlands.	15](#_Toc229055940)

[Table S5. Adjusted prevalence of having any COVID-19 related symptom during follow-up by ethnicity, the HELIUS COVID-19 substudy, May-November 2022, Amsterdam, the Netherlands. Sensitivity analysis among 982 participants reporting a past SARS-CoV-2 infection and/or testing SARS-CoV-2 antibody positive.	18](#_Toc229055941)

[Table S6. Determinants of PCC among HELIUS participants reporting a past SARS-CoV-2 infection or testing SARS-CoV-2 antibody positive, the HELIUS COVID-19 substudy, May-November 2022, Amsterdam, the Netherlands.	20](#_Toc229055942)

# Supplementary Methods

## Assessing COVID-19 related symptoms

Presence and duration of 15 COVID-19 related symptoms was measured using the following questions:

The following questions are about which complaints you had with this corona infection and for how long.

| Symptom | Did you experience this? | If yes, how long did the complaint last? | Still experiencing it? |
| --- | --- | --- | --- |
| 7.3 Mild fever (temperature between 37.5–38°C) | □ Yes / □ No | □ <1 week  □ 1–4 weeks  □ 1–3 months  □ 4–6 months  □ 7–12 months  □1–2 years  □ >2 years | □ Yes / □ No |
| 7.4 Fever of 38°C or higher | □ Yes / □ No | □ <1 week  □ 1–4 weeks  □ 1–3 months  □ 4–6 months  □ 7–12 months  □1–2 years  □ >2 years | □ Yes / □ No |
| 7.5 Cough | □ Yes / □ No | □ <1 week  □ 1–4 weeks  □ 1–3 months  □ 4–6 months  □ 7–12 months  □1–2 years  □ >2 years | □ Yes / □ No |
| 7.6 Sore throat or throat infection | □ Yes / □ No | □ <1 week  □ 1–4 weeks  □ 1–3 months  □ 4–6 months  □ 7–12 months  □1–2 years  □ >2 years | □ Yes / □ No |
| 7.7 Breathlessness or shortness of breath | □ Yes / □ No | □ <1 week  □ 1–4 weeks  □ 1–3 months  □ 4–6 months  □ 7–12 months  □1–2 years  □ >2 years | □ Yes / □ No |
| 7.8 Runny nose (e.g., sneezing, runny or blocked nose) | □ Yes / □ No | □ <1 week  □ 1–4 weeks  □ 1–3 months  □ 4–6 months  □ 7–12 months  □1–2 years  □ >2 years | □ Yes / □ No |
| 7.9 Muscle pain | □ Yes / □ No | □ <1 week  □ 1–4 weeks  □ 1–3 months  □ 4–6 months  □ 7–12 months  □1–2 years  □ >2 years | □ Yes / □ No |
| 7.10 Loss of smell and/or taste (longest-lasting complaint considered) | □ Yes / □ No | □ <1 week  □ 1–4 weeks  □ 1–3 months  □ 4–6 months  □ 7–12 months  □1–2 years  □ >2 years | □ Yes / □ No |
| 7.11 Reduced or no appetite | □ Yes / □ No | □ <1 week  □ 1–4 weeks  □ 1–3 months  □ 4–6 months  □ 7–12 months  □1–2 years  □ >2 years | □ Yes / □ No |
| 7.12 Fatigue / feeling very weak | □ Yes / □ No | □ <1 week  □ 1–4 weeks  □ 1–3 months  □ 4–6 months  □ 7–12 months  □1–2 years  □ >2 years | □ Yes / □ No |
| 7.13 Headache | □ Yes / □ No | □ <1 week  □ 1–4 weeks  □ 1–3 months  □ 4–6 months  □ 7–12 months  □1–2 years  □ >2 years | □ Yes / □ No |
| 7.14 Impaired concentration | □ Yes / □ No | □ <1 week  □ 1–4 weeks  □ 1–3 months  □ 4–6 months  □ 7–12 months  □1–2 years  □ >2 years | □ Yes / □ No |
| 7.15 Memory complaints | □ Yes / □ No | □ <1 week  □ 1–4 weeks  □ 1–3 months  □ 4–6 months  □ 7–12 months  □1–2 years  □ >2 years | □ Yes / □ No |
| 7.16 Confusion | □ Yes / □ No | □ <1 week  □ 1–4 weeks  □ 1–3 months  □ 4–6 months  □ 7–12 months  □1–2 years  □ >2 years | □ Yes / □ No |
| 7.17 Reduced ability to perform daily activities independently (self-reliance) | □ Yes / □ No | □ <1 week  □ 1–4 weeks  □ 1–3 months  □ 4–6 months  □ 7–12 months  □1–2 years  □ >2 years | □ Yes / □ No |
| 7.18 Hair loss | □ Yes / □ No | □ <1 week  □ 1–4 weeks  □ 1–3 months  □ 4–6 months  □ 7–12 months  □1–2 years  □ >2 years | □ Yes / □ No |
| 7.19 Other complaints (specify below) | □ Yes / □ No | □ <1 week  □ 1–4 weeks  □ 1–3 months  □ 4–6 months  □ 7–12 months  □1–2 years  □ >2 years | □ Yes / □ No |
| 7.19.1 Explanation for other complaints |  |  |  |

# Supplementary Figures

## Figure S1. Flowchart of the inclusion of HELIUS participants in the third visit of the COVID-19 substudy, Amsterdam, the Netherlands, May 17 - November 21, 2022.

Abbreviations: HELIUS Healthy Life in an Urban Setting; COVID-19 Coronavirus disease 2019; SARS-CoV-2 Severe Acute Respiratory Syndrome Coronavirus 2. In the first COVID-19 substudy visit, 2,496 individuals participated, of whom 2,088/2,496 attended the second visit and 1,505/2,496 attended the third visit. A total of 1,418 individuals participated in all three substudy visits. The HELIUS follow-up visit which took place between May 2019 and November 2022 was not included in this flowchart. Among those who participated in the first COVID-19 substudy visit 2,461/2,496, also attended the HELIUS follow-up visit.

## Figure S2. Radar plots presenting the proportion of each COVID-19 related symptom by ethnic group at month 3 and 12 by ethnicity, the HELIUS COVID-19 substudy, May-November 2022, Amsterdam, the Netherlands.

A. Proportion reporting a persistent COVID-19 related symptoms at 3 months after infection (n=851) B. Proportion reporting a persistent COVID-199 related symptoms at 12 months after infection (n=398). Abbreviations: *HELIUS* Healthy Life in an Urban Setting; *SARS-CoV-2* Severe acute respiratory syndrome coronavirus; *COVID-19* Coronavirus disease 2019.

# Supplementary Tables

## Table S1. Characteristics of the HELIUS third COVID-19 participants, by inclusion in the present study, the HELIUS COVID-19 substudy, May-November 2022, Amsterdam, the Netherlands (n=1505).

| **Characteristics** | **HELIUS participants in COVID-19 substudy visit 3 (n=1505)** | **HELIUS participants included (n=932)** | **HELIUS participants excluded (n=573)** |
| --- | --- | --- | --- |
|  | n (%) | n (%) | n (%) |
| **Sociodemographic factors** |  |  |  |
| **Ethnicity ^a^** |  |  |  |
| Dutch | 380 (25.2) | 229 (24.6) | 151 (26.4) |
| South-Asian Surinamese | 332 (22.1) | 195 (20.9) | 137 (23.9) |
| African Surinamese | 285 (18.9) | 170 (18.2) | 115 (20.1) |
| Ghanaian | 136 (9.0) | 53 (5.7) | 83 (14.5) |
| Turkish | 186 (12.4) | 143 (15.3) | 43 (7.5) |
| Moroccan | 186 (12.4) | 142 (15.2) | 44 (7.7) |
| **Age in years**, **median** **(IQR) ^a,^ ^b^** | 58.00 [49.00, 65.00] | 56.00 [46.00, 63.00] | 61.00 [54.00, 68.00] |
| <45 | 267 (17.7) | 204 (21.9) | 63 (11.0) |
| 45-54 | 321 (21.3) | 238 (25.5) | 83 (14.5) |
| 55-59 | 247 (16.4) | 138 (14.8) | 109 (19.0) |
| ≥60 | 670 (44.5) | 352 (37.8) | 318 (55.5) |
| **Sex ^a^** |  |  |  |
| Male | 642 (42.7) | 367 (39.4) | 275 (48.0) |
| Female | 863 (57.3) | 565 (60.6) | 298 (52.0) |
| **Education level ^a^** |  |  |  |
| No higher vocational or university education | 933 (63.2) | 580 (63.4) | 353 (62.9) |
| Higher vocational or university education | 543 (36.8) | 335 (36.6) | 208 (37.1) |
| Missing | 29 (1.9) | 17 (1.8) | 12 (2.1) |
| **Health factors** |  |  |  |
| **Smoking** **^c^** |  |  |  |
| Non smoker | 773 (51.8) | 473 (50.9) | 300 (53.4) |
| Current smoker | 288 (19.3) | 154 (16.6) | 134 (23.8) |
| Former smoker | 430 (28.8) | 473 (50.9) | 300 (53.4) |
| Missing | 14 (0.9) | 3 (0.3) | 11 (1.9) |
| **BMI ^c^** |  |  |  |
| Normal | 538 (37.3) | 315 (34.3) | 223 (42.7) |
| Overweight | 590 (40.9) | 385 (41.9) | 205 (39.3) |
| Obese | 313 (21.7) | 219 (23.8) | 94 (18.0) |
| Missing | 64 (4.3) | 13 (1.4) | 51 (8.9) |
| **CVD** **^c^** |  |  |  |
| No | 1406 (94.8) | 890 (94.8) | 516 (94.9) |
| Yes | 77 (5.2) | 49 (5.2) | 28 (5.1) |
| Missing | 22 (1.5) | 4 (0.4) | 18 (3.1) |
| **Asthma-COPD ^c^** |  |  |  |
| No | 1424 (94.9) | 887 (95.2) | 537 (94.4) |
| Yes | 77 (5.1) | 45 (4.8) | 32 (5.6) |
| Missing | 4 (0.3) | 0 (0.0) | 4 (0.7) |
| **Type 2 DM** **^c^** |  |  |  |
| No | 1314 (87.7) | 819 (88.2) | 495 (87.0) |
| Yes | 184 (12.3) | 110 (11.8) | 74 (13.0) |
| Missing | 7 (0.5) | 3 (0.3) | 4 (0.7) |
|  |  |  |  |
| **Hypertension ^c^** |  |  |  |
| No | 863 (57.6) | 563 (59.7) | 300 (54.1) |
| Yes | 635 (42.4) | 380 (40.3) | 255 (45.9) |
| Missing | 7 (0.5) | 0 (0.0) | 7 (1.2) |
| **Comorbidities ^c^** |  |  |  |
| No | 764 (51.5) | 503 (54.2) | 261 (47.0) |
| 1 or more | 719 (48.5) | 425 (45.8) | 294 (53.0) |
| Missing | 22 (1.5) | 4 (0.4) | 18 (3.1) |
| **Vaccination status ^d, e^** |  |  |  |
| Non vaccinated | 195 (13.0) | 162 (17.4) | 33 (5.8) |
| Vaccinated | 1309 (87.0) | 769 (82.6) | 540 (94.2) |
| Missing | 1 (0.1) | 1 (0.1) | 0 (0.0) |
| **SARS-CoV-2 antibody test result at previous study visit ^f^** |  |  |  |
| Negative at the second COVID-19 substudy visit | 1047 (69.6) | 598 (68.4) | 479 (88.4) |
| Positive at the first and/or second COVID-19 substudy visit | 458 (30.4) | 276 (31.6) | 63 (11.6) |
| Missing | 89 (5.9) | 58 (6.2) | 31 (5.4) |

Abbreviations: *HELIUS* Healthy Life in an Urban Setting; *COVID-19* Coronavirus disease 2019; *IQR* interquartile range; *SARS-CoV-2* Severe acute respiratory syndrome coronavirus; *BMI* Body mass index; *CVD* Cardiovascular disease; *COPD* Chronic obstructive pulmonary disease; *DM* Diabetes mellitus. ^a^ Measured at HELIUS baseline (2011–2015). ^b^ Age was recalculated for the third COVID-19 substudy visit. ^c^ Measured at HELIUS follow-up (2019-2021). ^d^ Measured during the third COVID-19 substudy visit (May-November 2022). ^e^ SARS-CoV-2 vaccination status was determined by the question “Which primary vaccinations have you received?”. ^f^ Having a positive SARS-CoV-2 antibody test result due to prior infection was defined as having a positive antibody test result from the first and/or the second COVID-19 substudy visits (visit 1: June-October 2020 or visit 2: November 2020-June 2021, before the COVID-19 vaccination was fully implemented in the Netherlands). Having a negative SARS-CoV-2 antibody test result due to prior infection was defined as having a negative antibody test result from the second COVID-19 substudy visit. Participant with an equivocal test result from the second COVID-19 substudy, with a negative test result from the first COVID-19 substudy visit who did not participate the second COVID-19 substudy visit are categorised as missing.

## Table S2. Adjusted prevalence estimates of having any COVID-19 related symptom during follow-up by ethnicity, the HELIUS COVID-19 substudy, May-November 2022, Amsterdam, the Netherlands. Main analysis among 932 participants reporting a past SARS-CoV-2 infection.

| Time | All | Dutch | South-Asian Surinamese | African Surinamese | Ghanaian | Turkish | Moroccan |
| --- | --- | --- | --- | --- | --- | --- | --- |
|  | aP (CI 95%)  (n/N) | aP (CI 95%)  (n/N) | aP (CI 95%)  (n/N) | aP (CI 95%)  (n/N) | aP (CI 95%)  (n/N) | aP (CI 95%)  (n/N) | aP (CI 95%)  (n/N) |
| Month 0 | 95% (93%-96%)  (886/932) | 96% (93%-98%)  (220/229) | 96% (93%-98%)  (188/195) | 94% (89%-96%)  (159/170) | 87% (75%-94%)  (46/53) | 97% (93%-99%)  (139/143) | 94% (89%-97%)  (134/142) |
| Month 1 | 44% (40%-48%)  (402/921) | 29% (23%-36%)  (65/224) | 51% (43%-58%)  (98/192) | 42% (34%-50%)  (71/169) | 26% (16%-40%)  (14/53) | 56% (48%-64%)  (79/141) | 53% (44%-61%)  (75/142) |
| Month 3 | 32% (28%-36%)  (271/851) | 17% (13%-24%)  (34/195) | 41% (34%-48%)  (74/182) | 31% (24%-39%)  (47/152) | 22% (13%-36%)  (11/49) | 40% (32%-49%)  (54/135) | 37% (29%-45%)  (51/138) |
| Month 6 | 32% (28%-36%)  (203/637) | 25% (18%-35%)  (26/102) | 41% (33%-49%)  (55/134) | 26% (19%-35%)  (31/119) | 17% (9%-31%)  (8/46) | 37% (29%-45%)  (44/119) | 33% (25%-42%)  (39/117) |
| Month 12 | 36% (31%-41%)  (142/398) | 30% (19%-44%)  (17/56) | 47% (36%-58%)  (39/83) | 30% (20%-41%)  (21/71) | 17% (7%-35%)  (5/29) | 41% (31%-52%)  (34/82) | 34% (24%-45%)  (26/77) |
| Month 24 | 32% (24%-41%)  (40/125) | 19% (8%-39%)  (5/26) | 56% (37%-74%)  (14/25) | 44% (5%-66%)  (8/18) | 0% (0%-0%)  (0/9) | 18% (7%-39%)  (4/22) | 36% (20%-55%)  (9/25) |

Abbreviations: *HELIUS* Healthy Life in an Urban Setting; *COVID-19* Coronavirus disease 2019; *aP* Adjusted Prevalence; *CI* Confidence interval; *n* Number; *N* Number. *n* is the number of symptomatic participants in each ethnic group at each time point, based on the reconstructed follow-up visits since infection (see methods). *N* is the number of individuals who did not reach their last reconstructed follow-up visit for each ethnic group at each time point (see methods). Of note, not all individuals had data until the 24 month and earlier reconstructed follow-up visits. This model is adjusted for age and sex.

## Table S3. Determinants of PCC among HELIUS participants - Univariable analysis, the HELIUS COVID-19 substudy, May-November 2022, Amsterdam, the Netherlands.

|  | **Univariable analysis**  **(n=819)** |
| --- | --- |
|  | OR (95% CI) |
| **Ethnicity ^a^** |  |
| Dutch | Ref |
| South-Asian Surinamese | 2.42 (1.59-3.67) |
| African Surinamese | 1.48 (0.95-2.30) |
| Ghanaian | 0.70 (0.35-1.38) |
| Turkish | 2.05 (1.31-3.21) |
| Moroccan | 1.90 (1.22-2.98) |
| **Age in years ^b^** |  |
| <45 |  |
| 45-54 | 1.67 (1.16-2.42) |
| 55-59 | 1.88 (1.24-2.85) |
| ≥60 | 1.23 (0.86-1.77) |
| **Sex** ^a^ |  |
| Male | Ref |
| Female | 2.08 (1.56-2.78) |
| **Education level** ^a^ |  |
| No higher vocational or university education |  |
| Higher vocational or university education | 0.50 (0.37-0.67) |
| **Employment status before infection ^c^** |  |
| Paid employed | Ref |
| Unemployed/student/retirement | 1.34 (0.99-1.81) |
| **Difficulty with Dutch language ^a, d^** |  |
| No | Ref |
| Yes | 1.25 (0.89-1.75) |
| **BMI ^e^** |  |
| Normal | Ref |
| Overweight | 1.42 (1.04-1.94) |
| Obese | 1.91 (1.33-2.73) |
| **Comorbidity ^e ,^ ^f^** |  |
| No | Ref |
| 1 or more | 1.20 (0.92-1.58) |
| **Vaccination status and calendar period of SARS-COV-2 infection ^c, g, h^** |  |
| Infected in the Pre-Alpha period | Ref |
| Unvaccinated and infected in the Alpha-Delta period | 1.17 (0.64-2.15) |
| Vaccinated and infected in the Alpha-Delta period | 1.17 (0.80-1.71) |
| Unvaccinated and infected in the Omicron period | 0.32 (0.19-0.54) |
| Vaccinated and infected in the Omicron | 0.32 (0.23-0.44) |

Abbreviations: *COVID* Coronavirus disease; *SARS-CoV-2* Severe acute respiratory syndrome coronavirus; ^a^ Measured at HELIUS baseline (2011–2015). ^b^ Age at the infection date. ^c^ Measured during the third COVID-19 substudy visit (May-November 2022) ^d^ For 211 Dutch participants, migration-related variables did not apply and were excluded from the analysis. ^e^ Measured at HELIUS follow-up (2019-2021). ^f^ Comorbidities include cardiovascular disease history, hypertension, diabetes, asthma/chronic obstructive pulmonary disease. ^g^ SARS-CoV-2 vaccination status was determined by the question “Which primary vaccinations have you received?”. Incomplete: received one dose of a vaccine other than Janssen, with or without subsequent infection; complete: received two doses of Pfizer, Moderna or AstraZeneca, ≥1 dose of Janssen, or had a past infection and subsequently received ≥1 dose of any vaccine (based on the guidelines of the Dutch government, reference: National Institute for Public Health and the Environment. COVID-19-vaccinatie uitvoeringsrichtlijn – version 4 December 2021. 2021. Available from: [https://lci.rivm.nl/richtlijnen/covid-19-vaccinatie. Accessed 29 April 2025](https://lci.rivm.nl/richtlijnen/covid-19-vaccinatie.%20Accessed%2029%20April%202025%20) ). Only two participants vaccinated incomplete, therefore categorised together with the complete group and named as vaccinated. ^h^ Calendar period of SARS-CoV-2 infection was categorised in 4 groups to reflect the dominant variant circulating in the Netherlands: pre-alpha period (January 2020- February 2021), alpha variant (March-June 2021), delta variant (July-December 2021) and omicron variant (January- October 2022). (Reference: National Institute for Public Health and the Environment Ministry of Health. Variants of the coronavirus SARS-CoV-2 2024 updated 16-10-2024. Available from: [https://www.rivm.nl/en/coronavirus-covid-19/current/variants. Accessed 29 April 2025](https://www.rivm.nl/en/coronavirus-covid-19/current/variants.%20Accessed%2029%20April%202025%20) ).

Table S4. Determinants of PCC among HELIUS participants - Multivariable analysis, the HELIUS COVID-19 substudy, May-November 2022, Amsterdam, the Netherlands. ^a^

|  | **Multivariable model 1**  **(n=819)** | | **Multivariable model 2**  **(n=585) ^b^** | | **Multivariable model 3**  **(n=609)^c^** | |
| --- | --- | --- | --- | --- | --- | --- |
|  | **aOR (95% CI)** | **P value** | **aOR (95% CI)** | **P value** | **aOR (95% CI)** | **P value** |
| **Ethnicity ^d^** |  | 0.002 |  | 0.217 |  | 0.002 |
| Dutch | Ref | - | Ref | - | NA | - |
| South-Asian Surinamese | 1.61 (1.02-2.56) | 0.043 | 0.96 (0.54-1.71) | 0.884 | Ref | - |
| African Surinamese | 0.92 (0.57-1.46) | 0.714 | 0.64 (0.36-1.15) | 0.137 | 0.54 (0.34-0.85) | 0.008 |
| Ghanaian | 0.40 (0.19-0.85) | 0.017 | 0.34 (0.13-0.89) | 0.028 | 0.26 (0.12-0.53) | <0.001 |
| Turkish | 1.51 (0.92-2.48) | 0.106 | 0.89 (0.47-1.70) | 0.728 | 0.86 (0.54-1.38) | 0.533 |
| Moroccan | 1.26 (0.74-2.15) | 0.386 | 0.79 (0.41-1.52) | 0.485 | 0.72 (0.45-1.15) | 0.170 |
| **Age in years ^e^** |  | 0.023 |  | 0.477 |  | 0.088 |
| <45 | Ref | - | Ref | - | Ref | - |
| 45-54 | 1.41 (0.96-2.08) | 0.083 | 1.25 (0.80-1.94) | 0.321 | 1.51 (0.99-2.29) | 0.054 |
| 55-59 | 1.94 (1.24-3.02) | 0.004 | 1.52 (0.89-2.60) | 0.125 | 1.75 (1.07-2.85) | 0.026 |
| ≥60 | 1.18 (0.79-1.78) | 0.421 | 1.25 (0.72-2.19) | 0.423 | 1.19 (0.75-1.89) | 0.461 |
| **Sex ^d^** |  | <0.001 |  | <0.001 |  | <0.001 |
| Male | Ref | - | Ref | - | Ref | - |
| Female | 2.35 (1.73-3.19) | <0.001 | 2.48 (1.71-3.60) | <0.001 | 2.73 (1.93-3.86) | <0.001 |
| **Education level ^d^** |  | 0.038 |  | 0.044 |  | - |
| No higher vocational or university education | Ref | - | Ref | - | - | - |
| Higher vocational or university education | 0.70 (0.50-0.98) | 0.038 | 0.65 (0.43-0.99) | 0.044 | - | - |
| **BMI ^f^** |  | 0.063 |  | - |  | 0.086 |
| Healthy weight | Ref | - | - | - | Ref | - |
| Overweight | 1.34 (0.96-1.89) | 0.089 | - | - | 1.37 (0.93-2.01) | 0.113 |
| Obese | 1.55 (1.06-2.28) | 0.024 | - | - | 1.58 (1.04-2.40) | 0.032 |
| **Vaccination status and calendar period of SARS-COV-2 infection ^g, i, j^** |  | <0.001 |  | <0.001 |  | <0.001 |
| Infected in the Pre-Alpha period | Ref | - | Ref | - | Ref | - |
| Unvaccinated and infected in the Alpha-Delta period | 1.09 (0.59-2.00) | 0.785 | 1.36 (0.53-3.48) | 0.526 | 1.10 (0.57-2.13) | 0.766 |
| Vaccinated and infected in the Alpha-Delta period | 1.31 (0.88-1.95) | 0.182 | 1.34 (0.80-2.22) | 0.266 | 1.24 (0.80-1.91) | 0.345 |
| Unvaccinated and infected in the Omicron period | 0.30 (0.17-0.53) | <0.001 | 0.35 (0.18-0.67) | <0.005 | 0.36 (0.20-0.66) | <0.005 |
| Vaccinated and infected in the Omicron | 0.33 (0.23-0.46) | <0.001 | 0.36 (0.23-0.54) | <0.001 | 0.37 (0.25-0.54) | <0.001 |
| **Sick leave due COVID-19 related symptoms in the first 4 weeks following infection ^g, h^** |  | - |  | <0.001 |  | - |
| No sick leave | - | - | Ref | - | - | - |
| <10 days | - | - | 1.86 (1.14-3.01) | 0.013 | - | - |
| 10-28 days | - | - | 5.24 (3.24-8.47) | <0.001 | - | - |

Abbreviations: *PCC* Post COVID-19 condition; *COVID-19* Coronavirus disease 2019; *SARS-CoV-2* Severe acute respiratory syndrome coronavirus 2; *HELIUS* Healthy Life in an Urban Setting; *aOR* Adjusted odds ratio; *CI* Confidence interval; *Ref* Reference category; *BMI* Body mass index. ^a^ All multivariable models were obtained by removing determinants with a P≥ 0.05 in a backward stepwise fashion using different set of potential determinants. Participants who have missing covariate values are excluded from analysis and ethnicity, age, and sex variables forced into all models. ^b^ Information on sick-leave was not available for 275 participants who were not working at the time of SARS-COV-2 infection. ^c^ For 211 Dutch participants, migration-related variables did not apply and were excluded from the analysis. ^d^ Measured at HELIUS baseline (2011–2015). ^e^ Age at the infection date. ^f^ Data on BMI was obtained from the follow-up visit. ^g^ Data on SARS-COV-2 variant, sick-leave due to SARS-CoV-2 related symptoms, and vaccination status were obtained from the 3^rd^ substudy visit. ^h^ Sick leave days variable is not evaluated in model 1, and model 3. ^i^ Calendar period of SARS-CoV-2 infection was categorised in 4 groups to reflect the dominant variant circulating in the Netherlands: pre-alpha period (January 2020- February 2021), alpha variant (March-June 2021), delta variant (July-December 2021) and omicron variant (January- October 2022). (Reference: National Institute for Public Health and the Environment Ministry of Health. Variants of the coronavirus SARS-CoV-2 2024 updated 16-10-2024. Available from: [https://www.rivm.nl/en/coronavirus-covid-19/current/variants. Accessed 29 April 2025](https://www.rivm.nl/en/coronavirus-covid-19/current/variants.%20Accessed%2029%20April%202025%20) ). ^j^ SARS-CoV-2 vaccination status was determined by the question ‘Which primary vaccinations have you received?’. Incomplete: received one dose of a vaccine other than Janssen, with or without subsequent infection; complete: received two doses of Pfizer, Moderna or AstraZeneca, ≥1 dose of Janssen, or had a past infection and subsequently received ≥1 dose of any vaccine (based on the guidelines of the Dutch government, reference: National Institute for Public Health and the Environment. COVID-19-vaccinatie uitvoeringsrichtlijn – version 4 December 2021. 2021. Available from: [https://lci.rivm.nl/richtlijnen/covid-19-vaccinatie. Accessed 29 April 2025](https://lci.rivm.nl/richtlijnen/covid-19-vaccinatie.%20Accessed%2029%20April%202025%20) ). Only two participants vaccinated incomplete, therefore categorised together with the complete group and named as vaccinated.

## Table S5. Adjusted prevalence of having any COVID-19 related symptom during follow-up by ethnicity, the HELIUS COVID-19 substudy, May-November 2022, Amsterdam, the Netherlands. Sensitivity analysis among 992 participants reporting a past SARS-CoV-2 infection and/or testing SARS-CoV-2 antibody positive.

| Time | All | Dutch | South-Asian Surinamese | African Surinamese | Ghanaian | Turkish | Moroccan |
| --- | --- | --- | --- | --- | --- | --- | --- |
|  | aP (CI 95%)  (n/N) | aP (CI 95%)  (n/N) | aP (CI 95%)  (n/N) | aP (CI 95%)  (n/N) | aP (CI 95%)  (n/N) | aP (CI 95%)  (n/N) | aP (CI 95%)  (n/N) |
| Month 0 | 89% (86%-92%)  (886/992) | 95% (92%-97%)  (220/231) | 93% (88%-96%)  (188/202) | 89% (84%-93%)  (159/178) | 53% (42%-63%)  (46/87) | 93% (88%-96%)  (139/149) | 92% (87%-96%)  (134/145) |
| Month 1 | 41% (36%-46%)  (402/981) | 29% (23%-35%)  (65/226) | 49% (42%-57%)  (98/199) | 40% (33%-48%)  (71/177) | 16% (10%-25%)  (14/87) | 54% (46%-62%)  (79/147) | 52% (43%-60%)  (75/145) |
| Month 3 | 30% (25%-35%)  (271/911) | 17% (12%-23%)  (34/197) | 39% (32%-47%)  (74/189) | 29% (23%-37%)  (47/160) | 13% (7%-22%)  (11/83) | 38% (31%-47%)  (54/141) | 36% (29%-45%)  (51/141) |
| Month 6 | 29% (25%-34%)  (203/697) | 25% (17%-35%)  (26/104) | 39% (31%-47%)  (55/141) | 24% (18%-33%)  (31/127) | 10% (5%-19%)  (8/80) | 35% (27%-44%)  (44/125) | 33% (25%-41%)  (39/120) |
| Month 12 | 31% (26%-37%)  (142/457) | 30% (19%-43%)  (17/57) | 43% (33%-54%)  (39/90) | 27% (18%-37%)  (21/79) | 8% (3%-18%)  (5/63) | 39% (29%-49%)  (34/88) | 33% (23%-44%)  (26/80) |
| Month 24 | 32% (24%-41%)  (40/127) | 19% (8%-39%)  (5/26) | 56% (37%-74%)  (14/25) | 44% (25%-66%)  (8/18) | 0% (0%-0%)  (0/11) | 18% (7%-39%)  (4/22) | 36% (20%-55%)  (9/25) |

Abbreviations: *HELIUS* Healthy Life in an Urban Setting; *COVID-19* Coronavirus disease 2019; *aP* Adjusted Prevalence; *CI* Confidence interval; *n* Number; N Number. *n* is the number of symptomatic participants in each ethnic group at each time point, based on the reconstructed follow-up visits since infection (see methods). *N* is the number of individuals who did not reach their last reconstructed follow-up visit for each ethnic group at each time point (see methods). Of note, not all individuals had data until the 24 month reconstructed and earlier follow-up visits. This model is adjusted for age and sex. Of the 992 participants, 932 reported infection and 60 did not but had a positive SARS-CoV-2 antibody test. Of the 60 participants who did not report infection despite a positive SARS-CoV-2 antibody test 2, 7, 8, 34, 6, and 3 were of Dutch, South-Asian Surinamese, African Surinamese, Ghanaian, Turkish, and Moroccan origin, respectively.

## Table S6. Determinants of PCC among HELIUS participants reporting a past SARS-CoV-2 infection or testing SARS-CoV-2 antibody positive, the HELIUS COVID-19 substudy, May-November 2022, Amsterdam, the Netherlands. ^a, b^

|  | **Multivariable model 1S**  **(n=867)** |
| --- | --- |
|  | aOR (95% CI) |
| **Ethnicity ^c^** |  |
| Dutch | Ref |
| South-Asian Surinamese | 1.42 (0.92-2.18) |
| African Surinamese | 0.85 (0.55-1.31) |
| Ghanaian | 0.16 (0.08-0.34) |
| Turkish | 1.29 (0.80-2.06) |
| Moroccan | 1.16 (0.70-1.92) |
| **Age in years ^d^** |  |
| <45 | Ref |
| 45-54 | 1.25 (0.86-1.81) |
| 55-59 | 1.64 (1.07-2.51) |
| ≥60 | 1.00 (0.68-1.47) |
| **Sex ^d^** |  |
| Male | Ref |
| Female | 2.12 (1.59-2.82) |
| **Education level ^c^** |  |
| No higher vocational or university education | Ref |
| Higher vocational or university education | 0.72 (0.53-0.99) |
| **BMI ^e^** |  |
| Healthy weight | Ref |
| Overweight | 1.35 (0.98-1.85) |
| Obese | 1.56 (1.08-2.24) |
| **Vaccination status and calendar period of SARS-COV-2 infection ^f, g^** |  |
| Infected in the Pre-Alpha period | Ref |
| Unvaccinated and infected in the Alpha-Delta period | 1.39 (0.77-2.51) |
| Vaccinated and infected in the Alpha-Delta period | 1.51 (1.03-2.22) |
| Unvaccinated and infected in the Omicron period | 0.44 (0.26-0.73) |
| Vaccinated and infected in the Omicron | 0.48 (0.35-0.66) |

Abbreviations: *PCC* Post COVID-19 condition; *COVID-19* Coronavirus disease 2019; *SARS-CoV-2* Severe acute respiratory syndrome coronavirus 2; *HELIUS* Healthy Life in an Urban Setting; *aOR* Adjusted odds ratio; *CI* Confidence interval; *Ref* Reference category; *BMI* Body mass index. ^a^ This analysis included 60 participants who did not report infection despite a positive SARS-CoV-2 antibody test. ^b^ This multivariable model was obtained by removing determinants with a P≥ 0.05 in a backward stepwise fashion using a set of potential determinants. Participants who have missing covariate values are excluded from analysis and ethnicity, age, and sex variables forced into all models. ^c^ Measured at HELIUS baseline (2011–2015). ^d^ Age at the infection date. ^e^ Data on BMI was obtained from the follow-up visit. ^f^ Data on dominant SARS-COV-2 variant circulating at the time of infection and vaccination status were obtained from the 3^rd^ substudy visit ^g^ Calendar period of SARS-CoV-2 infection was categorised in 4 groups to reflect the dominant variant circulating in the Netherlands: pre-alpha period (January 2020- February 2021), alpha variant (March-June 2021), delta variant (July-December 2021) and omicron variant (January- October 2022). (Reference: National Institute for Public Health and the Environment Ministry of Health. Variants of the coronavirus SARS-CoV-2 2024 updated 16-10-2024. Available from: [https://www.rivm.nl/en/coronavirus-covid-19/current/variants. Accessed 29 April 2025](https://www.rivm.nl/en/coronavirus-covid-19/current/variants.%20Accessed%2029%20April%202025%20) ). ^h^ SARS-CoV-2 vaccination status was determined by the question ‘Which primary vaccinations have you received?’. Incomplete: received one dose of a vaccine other than Janssen, with or without subsequent infection; complete: received two doses of Pfizer, Moderna or AstraZeneca, ≥1 dose of Janssen, or had a past infection and subsequently received ≥1 dose of any vaccine (based on the guidelines of the Dutch government, reference: National Institute for Public Health and the Environment. COVID-19-vaccinatie uitvoeringsrichtlijn – version 4 December 2021. 2021. Available from: [https://lci.rivm.nl/richtlijnen/covid-19-vaccinatie. Accessed 29 April 2025](https://lci.rivm.nl/richtlijnen/covid-19-vaccinatie.%20Accessed%2029%20April%202025%20) ). Only two participants vaccinated incomplete, therefore categorised together with the complete group and named as vaccinate.
